# The experiences and practices of oral health promotion for children in Cape Town: An exploratory descriptive qualitative study

**DOI:** 10.4102/phcfm.v18i1.5231

**Published:** 2026-01-31

**Authors:** Fathima Peerbhay, Robert Mash

**Affiliations:** 1 Department of Orthodontics, Faculty of Dentistry, University of the Western Cape, Cape Town, South Africa; 2 Department of Family and Emergency Medicine, Faculty of Medicine and Health Sciences, Stellenbosch University, Cape Town, South Africa

**Keywords:** oral health promotion, children, oral health, oral hygienists, dental caries, primary health care

## Abstract

**Background:**

In South Africa, oral diseases are a significant public health concern. The Western Cape has a high prevalence of dental caries, with rates increasing from 82% to 84% in 6-year-olds over a 13-year period. This study explored the experiences of oral hygienists, children and their parents to generate insights that can inform the design of a new approach to oral health promotion (OHP).

**Aim:**

This exploratory descriptive qualitative study was conducted within an interpretivist paradigm using semi-structured individual interviews with three groups of participants: oral hygienists, children aged 8–12 years and their parents.

**Setting:**

The study was conducted at the dental public health facilities in the Western Cape Metropole.

**Methods:**

The transcripts were thematically analysed using ATLAS.ti software and guided by the Ritchie and Spencer framework approach to thematic analysis.

**Results:**

Seven main themes were identified: oral health promotion approaches and effectiveness, professional development, barriers and challenges, children and parents’ oral health knowledge, attitudes and practices, parental influence and family dynamics, cultural and socio-economic considerations and engagement between oral hygienists, parents and children. Resource limitations within the public dental healthcare system had a negative effect on the hygienists’ ability to deliver effective OHP services.

**Conclusion:**

Although oral hygienists employ diverse methods, including practical demonstrations and interactive techniques, current OHP strategies remain inadequate to address the high burden of dental caries. These findings support the development of an alternative approach to OHP in this setting.

**Contribution:**

The study contributes to understanding the role of family dynamics in reducing the burden of oral diseases among children in the Western Cape.

## Introduction

**FIGURE 1:**
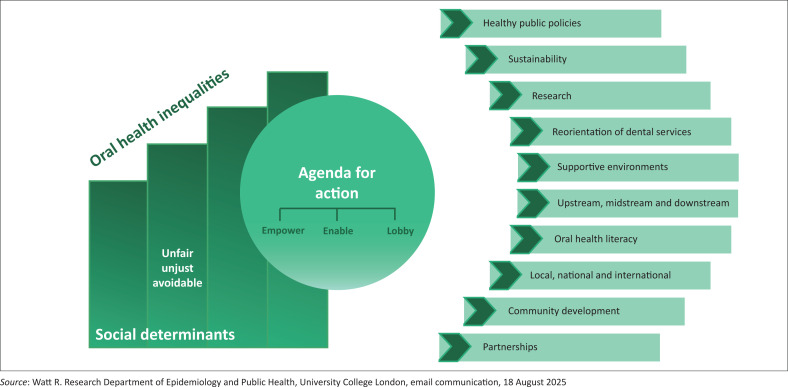
London Charter of Inequalities. Illustration used with permission from Richard Watt.

Oral health is an essential component of overall health and well-being and impacts an individual’s quality of life.^1^ The World Health Organization’s (WHO) Global Oral Health Meeting (Bangkok, November 2024) highlighted that oral diseases affect 3.5 billion people worldwide, posing a major public health challenge and highlighting the need to integrate oral health within the broader response to noncommunicable diseases.^2,3^ The most widespread oral condition is dental caries in permanent teeth.^4^ The consequences of dental caries extend beyond the oral cavity, leading to pain, infection, reduced quality of life, impaired nutrition and increased healthcare expenditure.^5^ Untreated dental caries in children impacts their growth, speech and school performance.^6^ Dental caries is an easily preventable disease and yet is still rife, disproportionately affecting disadvantaged and low socio-economic populations because of inequities in access to oral care, health education and preventive services.^7^ Social factors contribute to oral health inequalities that are ‘unfair, unjust and avoidable’ and the London Charter on oral health inequalities proposes an ‘agenda for action (Figure 1) on local, national and international levels to empower, enable and lobby’.^8^

The London Charter’s framework calls for coordinated efforts from local communities to international organisations, bringing together diverse stakeholders, including researchers, government officials, health professionals and the public.^8^ It advocates shifting dental care from a treatment-focused model to one that prioritises prevention and addresses the social conditions that create health disparities.^8^ The WHO’s global target (4,1) in relation to the integration of oral health into primary care is that ‘by 2030, 80% of countries have oral health care services generally available in primary health care facilities’.^3^ Globally, there is a transition from curative oral health strategies to preventive ones, as supported by the WHO Global Oral Health Action Plan 2023–2030, which promotes the incorporation of oral health into primary care systems (WHO).^3^ There is a need to include essential oral health services, such as promotion, prevention, early detection and treatment within universal health coverage (UHC) packages, prioritising delivery through primary health care (PHC) services (WHO).^3^ Oral health services should shift from curative to preventive care to empower communities in managing their oral health.^3^ The prevalence of oral diseases and dental caries is rising in the African and Middle Eastern Region, with the heaviest burden falling on the most disadvantaged communities.^9^ In South Africa, oral diseases remain a significant public health concern, particularly among children from low-income households.^10^ A review of dental caries prevalence and severity trends in South Africa found that among adolescents, the number of decayed, missing and filled teeth rose markedly between the ages of 12 years and 15 years.^11^ The Western Cape (South Africa) has a high prevalence of dental caries, with rates increasing from 82% to 84% in 6-year-olds over a 13-year period.^12^ This is an indication that current OHP strategies in the Western Cape are failing. Although the South African National Oral Health Strategy (SANOHS) (2024–2034) states that ‘a defined set of “Basic Oral Healthcare Services” should be part of the national PHC Package to be delivered at the district level’, dentists working at dental public health clinics reported that they were unable to provide the basic oral healthcare package because of insufficient resources.^13,14^ The SANOHS promotes the integration of oral health considerations into broader health policies and strategies.^13^ It also proposes that oral health should form an essential component of a comprehensive health promotion strategy across various service delivery platforms, including hospitals, community health centres, clinics, schools, orphanages and old age homes.^13^ However, service delivery is often hindered by resource constraints, workforce shortages and competing healthcare priorities, resulting in a continued emphasis on curative over preventive care.^13,14^ There is a compelling need for preventive oral health services to be prioritised over curative services to address the high burden of dental caries in South Africa.^11,12,15^ The SANOHS recommends oral health education and promotion, fissure sealants to 6- and 12-year-olds, mass fluoridation and toothbrushing at schools, as well as early childhood development centres.^13^ Oral hygienists are primary care providers positioned to deliver OHP to children and families and help them improve their oral health practices and oral health status.^16^ They play a pivotal role in delivering OHP within both clinical and community contexts. Their scope of practice includes preventive care, oral health education and the facilitation of behaviour change.^17^ Oral hygienists are uniquely positioned to engage directly with both children and their caregivers, ensuring consistent health messages with shared responsibility for oral health outcomes. Parents and caregivers are central to the development of children’s oral health practices.^18^ Their knowledge, beliefs and attitudes directly impact children’s habits, while children’s perceptions of oral health and dental experiences can shape their willingness to maintain healthy behaviours.^19^ Implementing structured educational programmes for parents can enhance their oral health knowledge and improve children’s oral health practice.^20^ Regular oral health education sessions with parents can help reinforce positive oral health behaviours and address new challenges that arise as children grow and develop.^20^ Effective OHP for children therefore requires an inclusive approach that addresses both the child and parent as active participants in the behaviour change process.^19^ Although global and national policy frameworks emphasise preventive oral health, there is limited South African research exploring the beliefs, attitudes and practices of oral hygienists, parents and children regarding OHP, particularly within the Western Cape Metropole. Understanding these perspectives is critical for designing interventions that are contextually relevant. The findings of this study have the potential to strengthen prevention-oriented programmes, enhance collaboration between oral health providers and families, and contribute to reducing the burden of oral diseases among children in the Western Cape.

## Research methods and design

### Study design

This exploratory, descriptive, qualitative study was conducted within an interpretivist paradigm, using semi-structured individual interviews with three groups of participants: oral hygienists, children aged 8–12 years and their parents.

### Setting

This study was conducted in Cape Town, Western Cape, South Africa. Cape Town is the second-largest city in South Africa and is characterised by significant cultural and linguistic diversity, with communities from multiple ethnic backgrounds. The three main spoken languages are IsiXhosa (37%), Afrikaans (31%) and English (28%).^21^ The Western Cape Health services are organised into Metro Health Services (MHS), Rural Health Services (RHS) and Tertiary or Specialised Hospitals. The MHS is divided into four substructures with two sub-districts each: Substructure 1 (Northern and Tygerberg sub-districts), Substructure 2 (Western and Southern sub-districts), Substructure 3 (Mitchells Plain and Klipfontein sub-districts), and Substructure 4 (Eastern and Khayelitsha sub-districts). The RHS are divided into five districts. Community health centres (CHCs) in the substructures are managed by a facility manager, who reports to the managing team of the substructure, with substructures reporting to the district.^22^ The CHCs provide primary health services, including oral health services, at a facility and community level.^22^ Dental clinics within CHCs are headed by a dentist, who reports to the facility manager and serves as part of the facility’s management team. Dentists, dental therapists and oral hygienists provide services in line with the basic package of care, according to their respective scopes of practice.^22^ While dentists and dental therapists primarily focus on clinical care, oral hygienists provide both clinical and community-based services in line with the Western Cape Road to Health report guidelines.^22^ The study was conducted at dental public health facilities in the Western Cape Metropole (WCM, City of Cape Town) district.

### Study population, sample size and sampling strategy

#### Oral hygienists

The study population comprised all 28 oral hygienists employed at public sector dental facilities in the Western Cape Metropole. Initial sampling aimed to include one oral hygienist from each of the eight sub-districts, with additional participants selected to ensure diverse representation. Participants were randomly selected using the sample function in R software. This process yielded an initial sample of 12 oral hygienists. The final sample size of 12 was determined by data saturation, with recruitment continuing until no new themes or insights emerged from the interviews.

#### Children and parents

A purposive sampling strategy was used to select children aged 8–12 years and their accompanying parents who attended appointments with the previously interviewed oral hygienists. Inclusion criteria for children included: ability to speak English, Afrikaans or IsiXhosa and being accompanied by a parent or legal guardian. Exclusion criteria included children with special needs or major systemic disease. A total sample of six children included a range of caries risk levels (low to high) as indicated by the oral hygienist. Six parents of the selected children were interviewed. Data saturation was again used to determine the final sample size.

### Data collection

The researcher conducted individual semi-structured interviews in English with oral hygienists, parents and children. The interviews were conducted at the selected dental public health clinics in the WCM after the oral hygienist had completed delivering OHP to the parent-and-child dyad. The interviews with the oral hygienists, parents and children were approximately 60 min, 45 min and 30 min long, respectively, and were audio-recorded with consent. A research assistant was present to assist the researcher if the participant was Afrikaans or IsiXhosa-speaking, as the researcher is fluent in English with a moderate command of Afrikaans. The interviews were conducted in a room at the dental clinic, and interviews for children were conducted in the presence of parents to minimise stranger anxiety. Parents were interviewed separately after their child’s interview, with the child waiting in a supervised area with dental personnel. Separate interview guides were developed for each participant group. The interview guides were designed by the researcher and developed from current evidence with similar objectives.^23,24,25^ The interview guides were piloted with a convenience sample of two oral hygienists and two parent-and-child dyads. The language of the interview guide for children was simplified further after the pilot study revealed that the children needed clarity regarding some terms in the original interview guide. The interview guides for the oral hygienists and parents did not need to be adjusted. These data were not included in the final dataset.

#### Oral hygienists

The oral hygienists’ opening question was ‘Tell me a little about your experience of delivering OHP in dental public health’, and specific topics to be explored were (Online Appendix 1):

- Prior experience of oral health behaviour change interventions.
- Beliefs about the effectiveness of the current OHP provided to children and parents.
- Attitudes towards developing person-centred oral health behaviour change interventions.
- Beliefs about the potential effect of person-centred approaches on children’s oral health status.

#### Children and parents

The opening question posed to the child as well as the parents was ‘why did you come to the clinic today’, and the following topics were then explored with both groups (Online Appendix 2 and Online Appendix 3):

- Beliefs about oral health and oral hygiene.
- Beliefs about the impact of sugar on oral health.
- Beliefs about the impact of dental disease on health and life.
- Beliefs about parental influence on oral health.
- Attitudes towards taking responsibility for oral health.
- Experience of OHP sessions with oral hygienists.

### Data analysis

Audio recordings were transcribed verbatim and checked for accuracy. The researcher analysed the transcripts thematically using ATLAS.ti software (Version 25) following the Ritchie and Spencer framework approach to thematic analysis.^26,27^ This approach was selected for its systematic, staged approach that enables rigorous and transparent thematic analysis. The purpose of thematic analysis was to extract relevant recurring elements from data and to use these patterns to provide insights into the topics of interest.^27^ The process followed is described next^27^:

- Familiarisation: Transcripts were read and re-read multiple times to become familiar with the interview data.
- Developing a coding framework: A coding index was created.
- Coding: The coding index was then applied to all the collected data.
- Charting: Reports were created that collated data with the same codes and categories in one document.
- Mapping and interpretation: The reports were interpreted for themes and sub-themes with illustrative quotations.

### Trustworthiness

The credibility of the analysis was improved by triangulation of data from the three different participant groups. In addition, member checking of the oral hygienists’ transcripts was conducted, and all oral hygienists confirmed that their transcripts were an accurate record of what they had said. In addition, the second author supervised the research process to ensure quality control of interviews, coding procedures and data interpretation. The four key issues were credibility, dependability, transferability and confirmability. The primary researcher is a professional dentist with over two decades of academic experience in Paediatric Dentistry. The researcher did not have any prior academic or clinical relationship with any of the oral hygienists interviewed, although they all studied at the same university, where they knew she held a senior lecturer position. This might have led to the oral hygienists providing socially desirable responses. Subjectivity of the data collection during the interviews was managed by the supervisor reviewing the first interview and advising that her prior training in Motivational Interviewing could possibly introduce bias, which raised the researchers’ awareness to help manage the interviews more objectively. The supervisor also assessed the quality of the coding index as well as the interpretation of the data.

### Ethical considerations

Ethical clearance to conduct this study was obtained from the University of Stellenbosch Health Research Ethics Committee (No. S20/03/061), and permission was granted by the Western Cape Department of Health (No. WC_202010_039). Written consent was requested from parents for their participation as well as for their children’s participation. Age-appropriate information was provided verbally to each child, and the researcher allowed the child an opportunity to ask questions before requesting their assent to participate in the research study in the presence of their parents.

## Results

Of the 12 facilities initially identified for the study, the Western Cape Department of Health granted authorisation to collect data at 8 facilities. However, facility managers at 2 of these authorised sites declined participation, resulting in a final sample of 6 facilities. Therefore, 6 oral hygienists were interviewed. The characteristics of the 18 respondents are shown in Table 1 and Table 2.

**TABLE 1:** Characteristics of oral hygienists.

Age of hygienist (years)	Gender of hygienist	Years of experience	Facility
59	Female	38	CDC 1
62	Female	30	CDC 2
44	Female	27	CDC 3
31	Female	9	CDC 4
47	Female	24	CDC 5
40	Female	20	CDC 6

CDC, community dental clinic.

**TABLE 2:** Characteristics of children and parents.

Age of child (years)	Gender of child	Parent	Facility
10	Female	Mother	CDC 1
10	Male	Mother	CDC 2
12	Male	Mother	CDC 3
8	Male	Mother	CDC 4
8	Male	Mother	CDC 5
9	Female	Father	CDC 6

CDC, community dental clinic.

Seven main themes were identified. The themes and sub-themes are presented in Table 3.

**TABLE 3:** Themes and sub-themes.

Themes	Sub-Themes
1. Oral health promotion approaches and effectiveness	1.1. Current approaches to OHP 1.2. Experiential learning and adaptive techniques 1.3. Effectiveness of OHP
2. Professional development	2.1. Previous training in OHP 2.2. Suggestions for future training
3. Barriers and challenges	-
4. Children and parents’ oral health knowledge, attitudes and practices	4.1. Oral health knowledge and practice (pain, discomfort, school disruption) 4.2. Brushing habits and routines 4.3. Responsibility for oral health
5. Parental influence and family dynamics	5.1. Parental role in children’s oral health 5.2. Defensive responses from parents 5.3. Family structures and caregiving responsibilities 5.4. Communication approaches with parents
6. Cultural and socio-economic considerations	6.1. Cultural beliefs and intergenerational transmission 6.2. Beliefs about primary teeth 6.3. Communication barriers 6.4. Socio-economic considerations
7. Engagement between oral hygienists, parents and children	7.1. Dietary advice 7.2. Engagement with oral hygienist

OHP, oral health promotion.

### Theme 1: Oral health promotion approaches and effectiveness

#### Sub-theme 1.1: Current approaches to oral health promotion

Oral hygienists highlighted the importance of using practical demonstrations when educating children about oral hygiene, as children learnt better when the procedure is modelled for them. This approach allowed children to develop practical skills under guidance and receive immediate feedback on their technique, which reinforced proper oral hygiene practices. They acknowledged the importance of including both parents and children in the OHP process, with parents viewed as primary decision-makers who could influence children’s oral health behaviours. Visual aids and age-appropriate explanations were regarded as valuable for teaching children oral health concepts. Half of the oral hygienists indicated that they used role models, storytelling and interactive activities to engage children. Oral hygienists reported that they adapted their OHP to the individual contexts and age of the children, using a variety of interactive techniques to improve the effectiveness of the OHP, for example, puppet shows:

‘When there is need, when there is plaque, calculus, mouth disease, my job is to teach them to do it, because at that age they can do it. I teach them in the chair with a toothbrush, with a mirror, I hold the mirror and say you brush and I see, and I teach them how to do it, I actually show them how it comes off, how it is shining. I then tell the parent, he is going to tell you I know how to brush, but you are still going to have nag, but you also have to help them to clean.’ (Oral hygienist, 59 years, CDC 1)

#### Sub-theme 1.2: Experiential learning and adaptive techniques

Oral hygienists mentioned that they adapted their approaches based on professional experience, learning what works through practice and modifying techniques to suit individual needs. All oral hygienists tailored their OHP approach to each child’s circumstances and unanimously endorsed practical demonstrations as their preferred teaching method, finding hands-on instruction more effective than theoretical explanations. They recognised the crucial role of involving both parents and children in oral health education, with parental involvement being a key determinant of success. Parents and oral hygienists mentioned that a lack of knowledge affects oral health practices. They further emphasised the importance of considering the context in which families live, noting that a lack of understanding of the living conditions of both parents and children can result in OHP efforts that are inappropriate and not tailored to the family’s specific needs:

‘Well, except for my obviously, my university degree, OK at that time it was a Diploma, sorry the correction, but we have also done development, skills development, patient management and behavioural management, things like that, that we do like that, so we do get short courses but I think most of the time it was learn while you work and that goes with experience. You see what works and what doesn’t work.’ (Oral hygienist, 59 years, CDC 1)

#### Sub-theme 1.3: Effectiveness of oral health promotion

Compliance with OHP varied significantly, with most hygienists reporting moderate adherence levels between 40% and 70%, and better compliance was observed among patients who attended regularly. Oral hygienists consistently reported alarmingly high rates of dental caries in the communities they served, indicating that current OHP strategies may be insufficient or ineffective:

‘I think about 50–70, I wouldn’t go above 70, because it is always the way you see the difference in their mouth when they come back for the follow up visit and you can see their attitude has changed. And then you get those kids that there is no change, and they come back because their parents tell them to. But I must say the compliance is very good, especially if you have the primary caregiver part of the promotion and the health.’ (Oral hygienist, 62 years, CDC 2)

### Theme 2: Professional development

#### Sub-theme 2.1: Previous training in oral health promotion

The oral hygienists demonstrated significant professional experience, with some having careers spanning decades. They engaged in continuing professional development to update their knowledge. While three oral hygienists mentioned receiving some training in client-centred approaches, they described it as brief and insufficient. All oral hygienists expressed a need for further training in psychological approaches to behaviour change. They reported that their OHP approaches had evolved over time through experience, shifting from simply providing information to aligning oral health instruction with the specific context of families. Hygienists identified gaps in their training related to addressing social determinants of oral health, suggesting a disconnect between theoretical knowledge and practical application:

‘Ja, maybe from a psychological point of view, if you guys can give us tips on how to help the patients, not bring across the message, so that the message that we give them, especially the parents, maybe give us ways to involve the parent so they feel it is actually their responsibility, because some of them feel it is not their responsibility, even though it is their child. If we can find a way psychologically to bring across the message, especially this type of parent we are working with here in this community, it is actually their responsibility, it is their child.’ (Oral hygienist, 40 years, CDC 6)

#### Sub-theme 2.2: Suggestions for future training

The findings of this study demonstrated strong support for a primary healthcare approach to oral health, with oral hygienists and parents advocating for multisectoral collaboration between dental professionals, education departments and broader community institutions. Both groups emphasised that effective OHP requires coordinated efforts across various channels, including schools, churches, community organisations and mass media, rather than relying solely on isolated clinical interventions:

‘It shouldn’t just be the oral hygienist who is doing it, I think the team and that is what I also liked about, like I need assistance, they have worked for a long time, they can talk to the parents as well, it must come from one source. If the team, the dentist, don’t leave it to the oral hygienist, we all work together, you know what I am saying? As a team, as opposed to saying this is, that is for the oral hygienist, that we all work together.’ (Oral hygienist, 62 years, CDC 2)

### Theme 3: Barriers and challenges

Resource limitations within the public dental healthcare system affected the availability of equipment, educational materials and staffing and had a negative effect on the hygienists’ ability to deliver effective OHP services. Although school-based oral health programmes had the potential to improve the oral health status of children, resource constraints limited their effectiveness:

‘Definitely resources. I must say the money I spend on resources; I mean that the stuff I used today, it is all my stuff, all the stuff that I had to, the booklets from Colgate, we had to buy it from Colgate, it was not given, we had to buy it. The models we had to buy from Colgate, so ja, it is a lot of effort from your side as well because when I got here, there was nothing, my room was like empty and then you have to start building up and getting a relationship.’ (Oral hygienist, 44 years, CDC 3)

### Theme 4: Children and parents’ oral health knowledge, attitudes and practices

#### Sub-theme 4.1: Oral health knowledge and practice

Children demonstrated a clear awareness of the physical pain and discomfort associated with oral health issues, particularly when describing their experiences of their teeth ‘becoming rotten’. Parents similarly acknowledged these physical effects and emphasised the wider implications for family well-being resulting from the deterioration of their children’s oral health. One child linked oral health problems to academic performance, while all the parents referred to the social and psychological consequences of having decayed teeth:

‘It affects my life when I have to go to school and do exams, and my tooth pains and my teacher has to send me home.’ (Child, male, 10 years, CDC 2)

‘Obviously, it can affect you negatively first of all, that is what I am saying. Lack of confidence, she is already shy so it would be even worse, you know what I am saying? She will stay indoors, first of all, she will not go out, not smile or even have a boyfriend or anything, with rotten teeth (laughing). No definitely, it will have a mental effect on her.’ (Parent, father, CDC 6)

#### Sub-theme 4.2: Brushing habits and routines

Children and parents demonstrated an understanding of basic oral hygiene but lacked comprehensive knowledge about preventive measures. There was variability in adherence to recommended brushing routines, with some children brushing only once a day and others twice daily. Parental engagement in children’s oral hygiene varied, with some actively involved in teaching and assisting, while others recognised a need for more education and support. Some parents also acknowledged their role in modelling oral health behaviours for their children:

‘I think that the parents must be more, how can I say, active in the, they must be more educated about the oral hygiene, and they must put more effort in, more time in teaching the children about oral hygiene.’ (Parent, mother, CDC 2)

#### Sub-theme 4.3: Responsibility for oral health

Both parents and children recognised that their responsibility for oral health changes as children grow older. Parents tended to take full responsibility for younger children’s oral hygiene but believed that this responsibility should be transferred to the children as they matured. Some parents viewed oral health as a shared responsibility between themselves and their children, ensuring that even as the child took more responsibility, parental supervision remained in place. Although parents recognised their role, they also faced challenges in managing children’s dietary preferences and oral hygiene behaviours:

[Interviewer] ‘Whose responsibility is it to brush your teeth?’ [Child] ‘It is my responsibility.’ (Child, 10 years, Male, CDC 2)

[Interviewer] ‘Whose responsibility is it about what he eats?’ [Parent] ‘It is my responsibility because if I give him one rand and he goes and buys sweets when he can actually buy a fruit, he needs to know to eat healthy.’ (Parent, mother, CDC 3)

### Theme 5: Parental influence and family dynamics

#### Sub-theme 5.1: Parental role in children’s oral health

Oral hygienists consistently identified parental attitude as perhaps the most significant determinant of successful OHP. Their experiences revealed that parental receptiveness directly correlated with improved oral health outcomes for children. All the oral hygienists interviewed believed that a collaborative approach involving both the parents and child during OHP was necessary. They identified the parents as being a crucial part of facilitating and maintaining oral health behaviour change in their children:

‘I think our biggest, I really our biggest, how do you say it, roadblock every day or our biggest challenge is just to get the patients to listen to us. I don’t think they listen., it is like I don’t know if it is boring for them or just not interesting but a lot of times it is just goes past them, you can talk and talk and talk and it just goes past them.’ (Oral hygienist, 44 years, CDC 3)

#### Sub-theme 5.2: Defensive responses from parents

Parental defensiveness was reported as a major barrier, often arising when parents interpreted OHP guidance as criticism rather than support. This defensive stance sometimes hindered productive engagement and reduced the likelihood of parents adopting recommended practices:

‘The challenge is their attitude when they perceive they have all the knowledge. if they come and tell me but they do brush and they have an electric toothbrush, then I know this is trouble, then whatever I say is not going to work, because they have already made up their mind. You can give them the best advice or whatever you want to do, beg, plead, cry even, but if they decide they are not here for talking, they can do whatever, you try to motivate, try to speak more, sometimes it takes a lot of time.’ (Oral hygienists, 59 years, CDC 1)

#### Sub-theme 5.3: Family structures and caregiving responsibilities

Hygienists observed the impact of grandparent-headed households and inconsistent caregiving arrangements, which disrupted continuity of care. These arrangements often meant that oral health routines were not consistently maintained, leading to poor oral health outcomes for children:

‘Also in this specific community, it is not only the parent that looks after the child, that is the difficult part also comes in with education, because most of the children with the grandmother or the aunt, the parent is in the picture, but say for instance the parent works, so now the child now stays with the grandparent in Bloekombos and the mother is staying in Scottsdene, I am just taking an example now. So, the child is moving from one house to another the whole time and then you will ask them, so do you brush your teeth at grandmother’s house, no I just brush it in the evening or only when I go to school.’ (Oral hygienist, 31 years, CDC 4)

#### Sub-theme 5.4: Communication approaches with parents

Some hygienists acknowledged that confrontational communication alienated parents. They recognised that adopting a supportive and empathetic communication style was more likely to encourage positive change in oral health behaviours:

‘Because I do sometimes struggle. Sometimes you get so, not, you get angry, what you see in the mouth and then immediately you jump on the parents because why? Why do you let your child look like this? What is happening? And then I can see, then I notice it a few times, OK this was not the right approach, because the parent is totally cut off because it is almost like you are disciplining them now and they are adults and it is their children, not your child, so I think to get an approach, to get the parent and the child, how to get a good approach to get them both involved but on their level and on the parent’s level.’ (Oral hygienist, 31 years, CDC 4)

### Theme 6: Cultural and socio-economic considerations

#### Sub-theme 6.1: Cultural beliefs and intergenerational transmission

Hygienists reported that some parents view oral health issues as hereditary and unavoidable, which discourages preventive care and influences their children’s beliefs. These intergenerational perceptions perpetuate cycles of poor oral health:

‘It is almost like they believe because their great grandfather had bad teeth, they have to have bad teeth and brushing is not going to solve the problem, so their gums are going to bleed, it doesn’t matter what you are going to do, my gums are going to bleed. I am not going to brush my teeth, and they had it and now I have it.’ (Oral hygienist, 44 years, CDC 3)

#### Sub-theme 6.2: Beliefs about primary teeth

Parents were perceived as undervaluing the importance of primary teeth, often opting for extractions over restorations. This belief reduced motivation for preventive care in early childhood:

‘I think with lots of parents they don’t think the primary teeth are so important. They don’t think it needs to be brushed. That oral health is part of your child’s general health and hygiene is a, there is a missing link there, you know, because the kids would be very neat otherwise, but oral health I think is still quite neglected, you know.’ (Oral hygienist, 62 years, CDC 2)

#### Sub-theme 6.3: Communication barriers

Language differences and unfamiliar terminology were considered as obstacles to effective OHP. Oral hygienists highlighted the need for culturally and linguistically tailored communication strategies:

‘Because I also believe you are also not going to take in information if you don’t get it, like how you would understand it. So sometimes with the Xhosa patients, they will understand English, but they will only hear it, they don’t really understand what you are trying to tell them, so that is a bit difficult with them. And to get someone then the whole time to try and interpret, you don’t always get that here, or to find someone, so you will then ask the security to please come and explain this important point to me, to the patient or to the mother, and then sometimes you will find the child understands the English but the mother doesn’t understand English at all, so to try and, so the language barrier I would say.’ (Oral hygienist, 31 years, CDC 4)

#### Sub-theme 6.4: Socio-economic considerations

Participants found that economic hardship often meant oral health was deprioritised in favour of basic needs. These financial pressures made it difficult for families to invest in preventive care or purchase oral hygiene products regularly:

‘There are other factors, you know, the socioeconomic, the other thing with the areas we are working in, the socioeconomic conditions play huge role in what the kids eat, do they have a toothbrush? You know, so that I think is key to sometimes things failing.’ (Oral hygienist, 62 years, CDC 2)

### Theme 7: Engagement between oral hygienists, parents and children

#### Sub-theme 7.1: Diet

Participants recalled dietary advice provided by hygienists, especially regarding sugar reduction. They noted that these discussions helped them understand the link between sugar consumption and dental problems:

‘There’s too much sugar in my porridge, I must only have one teaspoon.’ (Child, 8 years, CDC 4)

‘It was excellent, I learnt today, because I have another small one at home and my daughter as well, and she wants to just extract teeth, and I can tell her you can’t just lose your teeth, it affects your teeth, I learnt a lot from the oral hygienist.’ (Parent, mother, CDC 3)

#### Sub-theme 7.2: Engagement with oral hygienist

Children reported that oral hygienists played a crucial role in teaching them about oral hygiene, including brushing and flossing techniques. Parents reported learning new and important information about oral health and expressed commitment to improving their children’s oral hygiene habits. They also stated that they would begin actively checking and assisting with their children’s tooth brushing after these engagements:

‘She gave me the toothbrush, and she showed me how to brush my teeth morning, and I must do it and she showed me a better way and then she showed me how to do the other way so that my teeth, the plaque can stay away.’ (Child, female, 10 years, CDC 1)

Both children and parents rated their experiences with oral hygienists highly, often giving full marks. They valued the oral hygienists’ explanations, demonstrations and responsiveness to questions:

‘I would give her 10 out of 10 and she did all the explaining and answered the questions and there were things I also didn’t know, so I understand why it is important.’ (Parent, mother, CDC 5)

## Discussion

**FIGURE 2:**
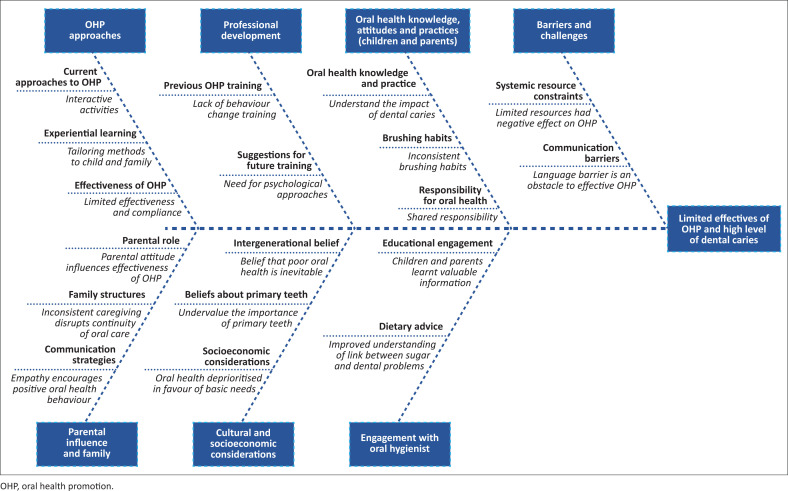
A summary of key findings.

The key findings are illustrated in Figure 2. The key findings are organised in a fishbone diagram into OHP approaches, professional development, children and parents’ oral health knowledge, attitudes and practices, barriers and challenges, parental influence and family dynamics, cultural and socio-economic considerations and engagement between oral hygienists, parents and children.

Oral hygienists reported that, in addition to providing oral health education information, they also relied on using practical demonstrations and interactive methods such as storytelling and puppet shows to educate children and parents to reinforce positive oral health behaviours. Interactive techniques have been shown to be more effective than conventional techniques in improving oral health behaviour in children.^28^ Many oral hygienists relied on experiential learning and adaptive practice, developing strategies through ‘trial and error’. Experiential learning has been shown to be an effective approach for enhancing oral health education, as confirmed in other studies.^29,30^ While this flexibility is beneficial, it also reflects limitations in formal training that motivate oral hygienists to resort to ‘trial and error’ strategies. The oral hygienists reported that their undergraduate training lacked behaviour change counselling. The psychology of behaviour change is well established in healthcare in general, and it is recommended that this evidence and theory be applied during OHP.^31,32^ It will, therefore, be useful for theory-based approaches to be included in the training of oral health professionals to improve the effectiveness of OHP.^32,33^ Adolescents from socially disadvantaged backgrounds are likely to benefit from theory-based approaches.^31^ Oral hygienists also expressed a need for training that contextualises OHP to the socio-economic circumstances of families. A comprehensive understanding of the intricate relationship between social determinants and oral health is vital for the development of targeted interventions that address health inequities and improve oral health outcomes.^34^ The literature confirms that socio-economic factors increase the incidence of dental caries, and there is a need for this to be considered in dental education.^35,36^ Strategies to reduce oral health inequalities should involve a major policy shift that prioritises addressing the structural and environmental factors driving chronic diseases.^37^ Resource constraints, such as limited equipment, a lack of educational materials and staffing, hindered hygienists’ capacity to provide effective OHP and this finding is supported by the current evidence.^38,39^ A global survey evaluated oral health prevention efforts 10 years after the 2007 WHO resolution, revealing major inequities between countries.^40^ Low- and middle-income countries have insufficient funding, workforce shortages and weak policies, making the WHO’s UHC goals difficult to achieve.^40^ Global oral health inequities persist a decade after WHO policy adoption, with resource-poor countries facing systemic barriers.^40^ Parents and children demonstrated an awareness of the pain, social stigma and academic disruption caused by poor oral health. Parents in this study also linked oral disease to broader family well-being, highlighting the psychosocial burden that oral health conditions impose on households. Other studies have also documented the detrimental effects that dental caries has on families.^41,42^ Children in this study generally understood the need for toothbrushing, but their toothbrushing practices varied. Some brushed once daily, while others managed toothbrushing twice daily. Parents reported varying levels of involvement, from active teaching and supervision, and they also recognised gaps in their own oral health knowledge. These findings align with evidence from a study examining parents’ experiences of toothbrushing with children,^43^ In this study, responsibility for oral hygiene was seen as evolving with age, with some parents of younger children assuming primary responsibility, while those with older children played a supervisory role. Parents recognised their responsibility but faced challenges in influencing children’s diet and oral hygiene practices. A study that explored parents’ experiences of toothbrushing with their young children concluded that parents in disadvantaged communities often struggled with their children’s resistant behaviour and faced stressful living conditions, leading them to take a passive supervisory role rather than actively managing their children’s brushing routines.^43^ The primary obstacles to effective parental supervision of brushing were a lack of behavioural management skills and challenging environmental circumstances that impacted family dynamics.^43^ Interestingly, many children considered themselves solely responsible for their oral health, reflecting a sense of independence, whereas parents viewed toothbrushing as a collaboration. Parental attitudes and receptiveness were cited by oral hygienists in this study as impacting the success of OHP. Other studies also found that parents are important facilitators responsible for shaping children’s oral health behaviours.^19,44,45^ Certain parental health behaviours, such as toothbrushing practices and frequency of consumption of sweet food, influence the oral health behaviours of young children.^44^ In addition, a defensive attitude can decrease the chances of parents implementing the suggested practices.^19,44,45^ Statistics South Africa reports that ‘millions of grandparents are stepping in to raise their grandchildren’, confirming the perspectives shared by the oral hygienists.^46^ Grandparents are an essential safety net for children in South Africa, but taking on this role places financial and emotional strain on older adults and may impact their attention to oral health.^46^ Oral hygienists in this study observed that a supportive communication style with parents was more effective in contributing to positive changes in oral health behaviours of their children. Parents face various challenges in raising children and an empathic, collaborative approach where parents are viewed as ‘knowledgeable partners’ is important in addressing oral health in children.^47^ This study reports that parents viewed poor oral health as hereditary and inevitable, an attitude termed fatalism, which is defined as the belief that events are fixed and beyond human control.^48^ Fatalism, although documented in relation to oral health, has limited evidence.^48,49^ One study found that parents with low confidence in managing oral health were more likely to endorse such beliefs.^50,51^ Furthermore, cultural beliefs influence oral health beliefs and oral health behaviour, and it is imperative that in multicultural populations, oral health instructions are tailored and adapted to the specific contexts of each cultural group.^52^ Dental professionals require improved cultural awareness and interpersonal communication abilities to better serve diverse patient populations.^52^ Oral hygienists in this study reported that parents in the communities they work in did not believe that primary teeth were important and opted to have decayed teeth extracted rather than restored. There is a need for parents to be educated about the importance of maintaining primary teeth, preventing and managing dental caries in children, and understanding treatment options available to improve children’s oral health outcomes.^50,51^ Oral hygienists emphasised the importance of communication strategies tailored to cultural and linguistic contexts in this study. Effective communication is vital for reducing patient anxiety, preventing misunderstandings and enabling positive relationships.^53^ Regular assessment and improvement of communication skills can enhance professional satisfaction, consultation efficiency and patient outcomes.^53^ Financial constraints were cited by oral hygienists as limiting families’ ability to purchase oral hygiene products, such as toothbrushes and toothpaste. Children from lower-income families experience significantly worse dental health, including more tooth decay and reduced quality of life.^54^ There are multiple contributing factors, such as parents’ education levels, financial resources, access to healthy food and community environments.^54^ Socio-economic disadvantages in early childhood can create lasting oral health problems that persist throughout life.^54^

### Strengths and limitations

The strength of the study is that the sample size was sufficient, as data saturation was reached and supported by a clear, auditable trail of data collection and analysis. In addition, transferability was addressed by having an in-depth description of the setting and people interviewed, and the findings are supported by the current evidence.

Although all the oral hygienists in this study were female, this does not represent a limitation as the selection was randomised. This sample reflects the current gender profile of the profession in South Africa, where most oral hygienists are female.^55^

### Implications

There is a need to develop new approaches to oral health behaviour change counselling to enhance engagement and motivation of parents and children to adopt healthier behaviours when they attend dental clinics. These interventions could incorporate psychological theory and person-centred approaches, such as motivational interviewing. Approaches should be family-oriented and recognise diverse family structures, including grandparent-headed households, and provide appropriate support for consistent caregiving.

## Conclusion

The findings reveal a complex interplay of factors that influence OHP in disadvantaged communities. This study demonstrates that while oral hygienists employ diverse and adaptive approaches, including practical demonstrations and interactive techniques, current strategies remain insufficient to address the high burden of dental caries. This research highlights the important role of parental attitudes and engagement in determining successful OHP, while also revealing significant cultural beliefs and socio-economic factors that influence oral health practices. The findings highlight the importance of addressing social determinants of health in OHP strategies. Communication barriers, intergenerational transmission of fatalistic beliefs about oral health, and economic hardship create substantial obstacles to effective preventive care. The findings of this study could inform the design of a new approach to OHP in this context.
